# Supplementary material for: Gpr149 is involved in energy homeostasis in the male mouse
Source: PeerJ. 2024 Jan 25;12:e16739. doi: 10.7717/peerj.16739 (PMC10822134; doi:10.7717/peerj.16739)
Supplement: Supplemental Information 1 [file peerj-12-16739-s001.docx]

| **Mice probes** | **Sequence** |
| --- | --- |
| Gpr149 null allele 5’ Guide ribonucleotides sequence | 5’- UUAUAACUGGUCACCUAUGUGUUUUAGAGCUAUGCU -3’ |
| Gpr149 null allele 3’ ribonucleotide sequence | 5’- UUGGUAGUUAACGAGACCCCGUUUUAGAGCUAUGCU-3’ |
| Null allele genotyping probes | 5’-GCTGCTTGTAATGTGTGCAGAGAG -3’  5’-GTCTACTCATGGCAGACCAAAGTAATGG-3’  5’- GTCTCTTGGTGCTAGAGATGGGTG-3’ |
| Cre-P2A-Gpr149 guide | 5’-AAGUCAUAAUUCUACGGAGAGUUUUAGAGCUAUGCU-3’ |
| **Taqman assays** | **Cat#** |
| Gpr149 | Mm00805216_m1 |
| 18s | Hs99999901_s1 |
| **RNAscope probe** | **Target region** |
| Gpr149 probe | 1436 – 2360 |
