# Supplementary figures and images for: Gpr149 is involved in energy homeostasis in the male mouse
Source: PeerJ. 2024 Jan 25;12:e16739. doi: 10.7717/peerj.16739 (PMC10822134; doi:10.7717/peerj.16739)

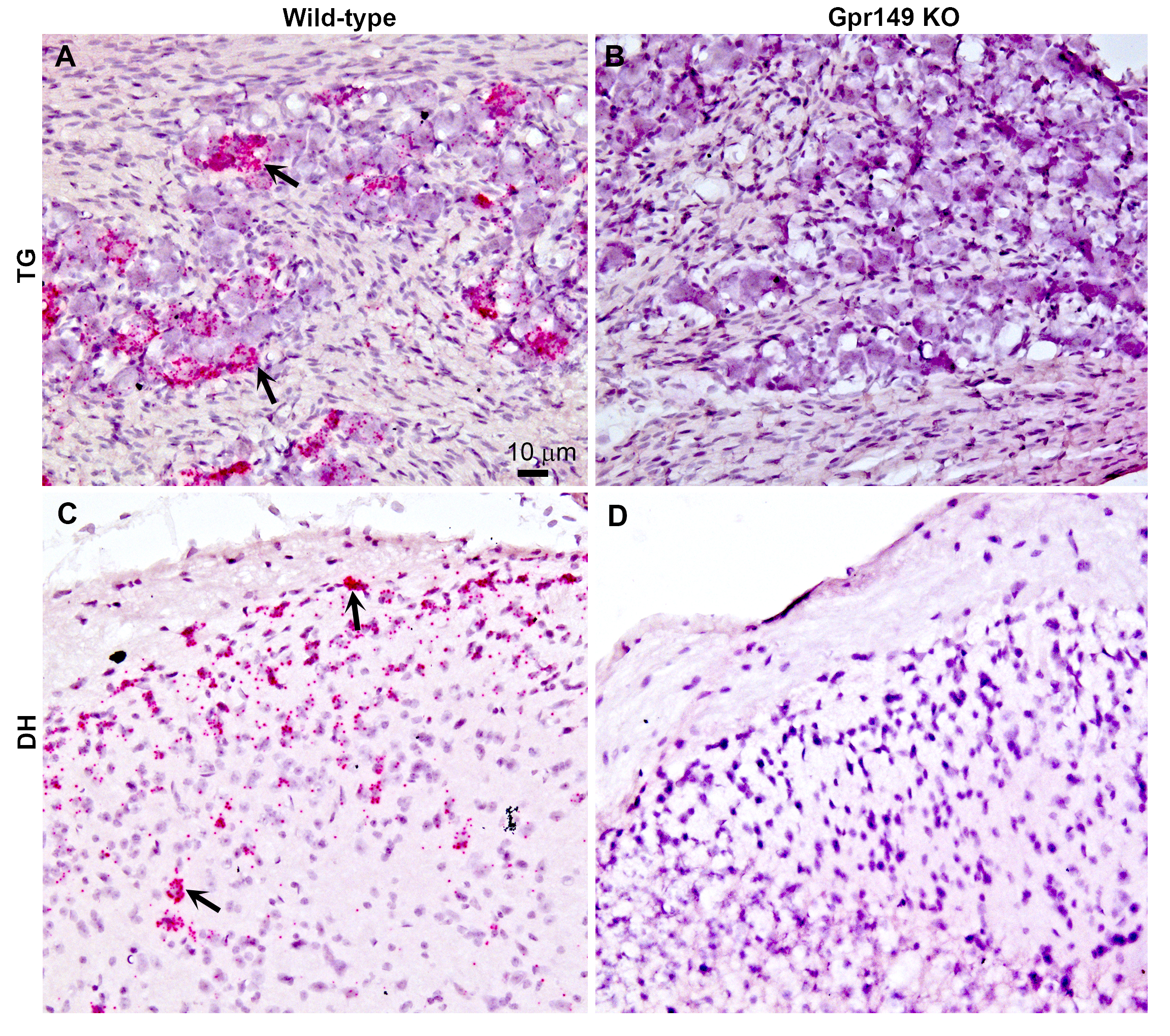

Supplement: Supplemental Information 4 — Chromogenic RNAscope ISH was used to assess Gpr149 expression (red dots) in wildtype (left) vs Gpr149−/− mice (right). Tissues were counterstained with hematoxylin and imaged with bright-field microscopy. (A) In the wild type mice, robust Gpr149 signals are observed in the trigeminal ganglion (TG) and (C) the dorsal horn (DH) of the spinal cord. Black arrows indicate representative Gpr149-positive cells. (B and D) In the Gpr149−/− mouse, TG and DH are completely devoid of signals. Scale bar in A applies to the entire plate. [file peerj-12-16739-s004.jpg]
